# Prediction of Major Depressive Disorder Following Beta-Blocker Therapy in Patients with Cardiovascular Diseases

**DOI:** 10.3390/jpm10040288

**Published:** 2020-12-18

**Authors:** Suho Jin, Kristin Kostka, Jose D. Posada, Yeesuk Kim, Seung In Seo, Dong Yun Lee, Nigam H. Shah, Sungwon Roh, Young-Hyo Lim, Sun Geu Chae, Uram Jin, Sang Joon Son, Christian Reich, Peter R. Rijnbeek, Rae Woong Park, Seng Chan You

**Affiliations:** 1 Department of Biomedical Informatics, Ajou University School of Medicine, Suwon 16499, Korea; jshsh7553@ajou.ac.kr; 2 Real World Solutions, IQVIA, Cambridge, MA 02139, USA; kristin.kostka@iqvia.com (K.K.); christian.reich@iqvia.com (C.R.); 3 Department of Medicine, School of Medicine, Stanford University, Stanford, CA 94305, USA; jdposada@stanford.edu (J.D.P.); nigam@stanford.edu (N.H.S.); 4 Department of Orthopaedic Surgery, College of Medicine, Hanyang University, Seoul 04763, Korea; estone96@gmail.com; 5 Department of Internal Medicine, Kangdong Sacred Heart Hospital, Hallym University College of Medicine, Seoul 05355, Korea; doctorssi@kdh.or.kr; 6 Department of Psychiatry, Ajou University School of Medicine, Suwon 16499, Korea; dongyun909@gmail.com (D.Y.L.); sjsonpsy@ajou.ac.kr (S.J.S.); 7 Department of Psychiatry, College of Medicine, Hanyang University, Seoul 04763, Korea; swroh@hanyang.ac.kr; 8 Division of Cardiology, Department of Internal Medicine, College of Medicine, Hanyang University, Seoul 04763, Korea; mdoim@hanyang.ac.kr; 9 Department of Industrial Engineering, Hanyang University, Seoul 04763, Korea; sgchae@psm.hanyang.ac.kr; 10 Department of Cardiology, Ajou University School of Medicine, Suwon 16499, Korea; statery@aumc.ac.kr; 11 Department of Medical Informatics, Erasmus Medical Center, 3015 GD Rotterdam, The Netherlands; p.rijnbeek@erasmusmc.nl; 12 Department of Biomedical Sciences, Ajou University Graduate School of Medicine, Suwon 16499, Korea

**Keywords:** adrenergic beta-antagonists, depressive disorder, machine learning, cardiovascular diseases

## Abstract

Incident depression has been reported to be associated with poor prognosis in patients with cardiovascular disease (CVD), which might be associated with beta-blocker therapy. Because early detection and intervention can alleviate the severity of depression, we aimed to develop a machine learning (ML) model predicting the onset of major depressive disorder (MDD). A model based on *L*1 regularized logistic regression was trained against the South Korean nationwide administrative claims database to identify risk factors for the incident MDD after beta-blocker therapy in patients with CVD. We identified 50,397 patients initiating beta-blockers for CVD, with 774 patients developing MDD within 365 days after initiating beta-blocker therapy. An area under the receiver operating characteristic curve (AUC) of 0.74 was achieved. A history of non-selective beta-blockers and factors related to anxiety disorder, sleeping problems, and other chronic diseases were the most strong predictors. AUCs of 0.62–0.71 were achieved in the external validation conducted on six independent electronic health records and claims databases in the USA and South Korea. In conclusion, an ML model that identifies patients at high-risk for incident MDD was developed. Application of ML to identify susceptible patients for adverse events of treatment may serve as an important approach for personalized medicine.

## 1. Introduction

Incidence of depression in patients with cardiovascular disease (CVD) is higher than that in healthy individuals [1]. Depression following CVD has been reported to be associated with mortality and new cardiovascular events [2,3]. The severity of depression and poor prognosis have a directly proportional relationship [1]. Furthermore, early diagnosis and intervention for depression in patients with CVD alleviate the disease severity and eventually benefit treatment outcome [4,5]. However, clinical predictors of incident depression following CVD are not well established [6].

Studies have suggested that subsequent depression or mood disturbance after CVD might be associated with beta-blocker therapy [7,8,9]. Beta-blockers are a widely prescribed drug for CVDs, including hypertension, myocardial infarction, coronary arteriosclerosis, angina pectoris, cardiac arrhythmia, and heart failure [10]. Nonetheless, the use of beta-blockers in patients with stable coronary artery disease, myocardial infarction, heart failure with preserved ejection fraction, or hypertension has been challenged due to paucity of evidence about the benefit of beta-blockers in these patients [11,12,13,14]. Unnecessary prescription of medications to susceptible patients may violate the so-called ‘First, do no harm’ injunction [15]. Risk stratification of patients with beta-blockers for subsequent depression may reduce drug-related morbidity, which is in line with personalized medicine [16].

Machine learning (ML) is widely used to solve prediction problems in medicine. Existing depression prediction models have mostly focused on limited socio-psychological factors and medical histories [17,18]. By contrast, ML can handle a large number of variables through a data-driven approach [19], so that developing reproducible machine-learning algorithms and validating the developed algorithms using heterogeneous external data sets has been demanding. Reps et al. proposed a standardized machine-learning framework to generate and evaluate a clinical prediction model that leverages standardized clinical databases to overcome this daunting challenge [20].

Therefore, we aimed to develop a robust prediction model that stratifies patients at risk of incident major depressive disorder (MDD) after using beta-blockers for CVD based on the standardized framework.

## 2. Materials and Methods

### 2.1. Data Source

We developed a prediction model using the National Health Insurance Service-National Sample Cohort (NHIS-NSC) database, South Korea [21]. This database is generated from claims of South Korea’s national health insurance. It is designed to represent the general population of South Korea by systematical sampling from all eligible insured individuals. In 2002, approximately one million individuals, which is equivalent to 2.2% of the South Korean population, were sampled and followed up for 11 years. The database covers information about age, sex, diagnosis, drugs, and procedures.

External validation was conducted on six electronic health records (EHR) and claims databases from the USA and South Korea. The databases from the USA include two EHR databases called IQVIA US Ambulatory Electronic Medical Record (EMR) and the STAnford medicine Research data Repository (STARR)—Observational Medical Outcomes Partnership (OMOP), and one claims database called IQVIA OpenClaims. The databases from South Korea include EHR databases from three tertiary teaching hospitals, Ajou University Hospital, Hanyang University Hospital, and Kangdong Sacred Heart Hospital.

The IQVIA US Ambulatory EMR database is composed of longitudinal, de-identified EHR originating from ambulatory clients spanning from 2006 to 2020. The database covers more than 40 million patients. IQVIA Open Claims database consists of open, pre-adjudicated medical (inpatient and outpatient) and pharmacy claims from 2013 to 2020. These data cover more than 200 million unique patients. The STARR, a clinical data warehouse, contains live Epic data from various hospitals. STARR-OMOP contains EHR data for more than 3 million patients from 2008 to 2020 [22]. More detailed information about the databases from the USA is available in Supplementary Table S1.

Ajou University Hospital database contains medical records of approximately 3 million inpatients and outpatients who visited between 1994 and 2018. Hanyang University Hospital database includes approximately 1.7 million inpatients and outpatients who visited between 2001 and 2018. Kangdong Sacred Heart Hospital database includes approximately 1.1 million inpatients and outpatients who visited between 1986 and 2019.

All databases had been converted to a standardized format called the OMOP common data model (CDM) [23]. Regional of institutional drug and diagnosis codes were converted to standardized OMOP vocabulary to provide interoperability between databases using different code systems, which has been developed and maintained by an international collaborative initiative, Observational Health Data Sciences and Informatics (OHDSI) [24].

### 2.2. Design

#### 2.2.1. Study Population and Outcome

Patients who were prescribed beta-blockers for a continuous exposure period of 30 days or more, were enrolled in the cohort. If the prescription interval for a particular drug is less than 30 days, it was regarded as continuous exposure. The first prescription date during a continuous exposure period is considered as the index date of a subject enters the cohort. Only beta-blockers used for CVD were included. We required patients to have a diagnosis record of hypertension, myocardial infarction, coronary arteriosclerosis, angina pectoris, or heart failure, within 3 days before and after the index date. Patients with a prescription history of antidepressants, or a diagnosis history of schizophrenia or depressive disorder, at any time before the index date were excluded. The patient’s age had to be 18 years or older on the index date. In addition, patients without 365 days of observable period prior to the index date were excluded to ensure that the index event was the first beta-blocker prescription longer than 30 days.

We defined outcome event as the onset of MDD within 365 days after the index date. Only the first MDD diagnosis within the follow up period was used as an outcome event. Patients without an observable period of 365 days after the index date were excluded to ensure that patients with non-occurrence of the outcome event are event-free.

Target cohort and outcome events were extracted using clinical data from claims and EHR databases converted to OMOP-CDM. The list of concepts and logics utilized to define the target cohort and outcome events are provided in Supplementary Table S2.

#### 2.2.2. Variables and Analysis

Variables to train the prediction model were generated from clinical data for 365 days of observable time prior to the index date. Clinical data include sex, age, diagnosis, prescription, and procedure. Furthermore, diagnosis covers various categories of disease such as cardiovascular diseases, mental disorders, and common chronic diseases. Clinical data were converted to binary format (yes or no), which has high usability for training the ML model. Variables were coded “no” for non-occurrence of a particular record. Exceptionally, age is used as continuous variable. Through this process, a total of 10,004 variables were generated.

The baseline characteristics of the study population with outcome, and without outcome, were compared. Mean age was calculated based on the index date, and age was also grouped by 10-year interval. Beta-blocker indication was derived from the records of CVD for three days before and after the index date. Other baseline characteristics of diagnoses and drugs were extracted from the history of 365 days prior to the index date. History of common chronic diseases, mental disorders, nutritional disorders, and medications associated with CVD and immunosuppressive therapy in the year prior to the index date, were compared. Differences of categorical variables between outcome and non-outcome groups were evaluated using the chi-squared test. When the frequency of categorical variables is less than 5, the Fisher’s exact test was used. The *t*-test was used to compare continuous variables. Statistical significance was defined as a *p*-value of <0.05 in a two-tailed test.

We investigated *L*1 regularized logistic regression, random forest, and gradient boosting machine as candidate algorithms to be used in the prediction model. These algorithms are widely used and are suitable for solving classification problems, such as the prediction of clinical outcomes [25]. Using default settings in each algorithm, we built three models using the NHIS-NSC claims database, for the comparison of performance. We found that the performances of three models were similar (area under the receiver operating characteristic curve (AUC) range, 0.67–0.69). However, the *L*1 regularized logistic regression algorithm significantly reduced the number of variables compared to other methods (58 versus 121–2597). The details of default algorithm settings and the results are provided in Supplementary Table S3. Considering that the need for more data in the clinical field is directly associated with increased cost, the prediction model is desirable for using fewer variables. Also, previous studies have demonstrated the usefulness of regression-based algorithms in clinical prediction compared with other modern ML algorithms [25,26].

Thus, the prediction model was developed based on the logistic regression method with *L*1 regularization. Logistic regression was used, because the variables used to train the model were binary. *L*1 regularization is a data-driven algorithm selecting the most predictive variables from numerous variables converted from clinical data. This enabled regression to be applied to extremely high-dimensional data, which have variables larger than sample size. Moreover, regularization reduces overfitting induced by including the training data-specific association in the model.

The model was trained against the NHIS-NSC database. The study population who met the inclusion and exclusion criteria was randomly split into 80% of the training set and 20% of the validation set. The training set was again randomly split into five groups, and the optimal parameters were derived for each group. Among them, the parameter of the best performed model was chosen. Then, the model was fitted using whole training set with the chosen parameters. This helps in suppressing the overfitting and retaining the generality across the databases. For the evaluation of model performance, AUC, sensitivity, and specificity were calculated. Furthermore, external validation of the developed model was conducted against six EHR and claims databases to demonstrate generalizability and to identify the possibility of overfitting.

Two additional analyses were conducted to improve understanding of the development data and the model. First, we investigated the distribution of beta-blockers in the development database by molecular type and year. The prescriptions were counted by year, from 2003 to 2012. Also, the numbers of beta-blocker prescriptions were counted by selectivity and lipophilicity. Furthermore, we calculated the incidence of MDD among patients with and without strong predictors.

This study followed the Transparent Reporting of a Multivariable Prediction Model for Individual Prognosis or Diagnosis (TRIPOD) reporting guideline for prediction algorithm validation [27]. All development of the prediction model and statistical analysis were carried out with R 3.6, and the framework including packages followed the patient-level prediction from the OHDSI community [20]. A dedicated R package to validate and apply the developed prediction model has been published in GitHub (https://github.com/ohdsi-studies/MddAfterBbValidation). The institutional review board (IRB) at Ajou University Hospital, Suwon, Korea, approved this study (IRB approval number: AJIRB-MED-MDB-20-382, AJIRB-MED-EXP-20-390).

## 3. Results

### 3.1. Baseline Characteristics

There were a total of 50,397 beta-blocker users who met the inclusion and exclusion criteria in the training database. Among them, 774 patients developed MDD within the following 365 days from the index date. Incident rate was 1.5%. Basic characteristics of the study population with outcome, and non-outcome are listed in Table 1.

Mean age of the outcome group and non-outcome group was 61.2 (standard deviation = 12.9) and 58.7 (standard deviation = 13.1) respectively, with no statistical significance. When the patients were grouped by age at 10-year intervals, we observed that patients in their 20s have a high incidence of outcome, especially in males. Thereafter, the incidence rate fell sharply and again gradually increased with age. The number of patients and their outcome incidence by age groups are presented in Supplementary Table S4. The proportion of males in the outcome group was 38.5%, which is significantly lower than 51.4% in the non-outcome group. The most frequent CVD in both outcome and non-outcome group was hypertensive disorder, 93.8% and 95.4% respectively (*p* = 0.050). Among CVD that showed statistical significance, angina pectoris and heart failure showed a higher proportion in the outcome group (*p* < 0.001, *p* = 0.003) and coronary arteriosclerosis was higher in non-outcome group (*p* = 0.021). Prevalence of chronic lung disease, stroke, and Alzheimer’s disease was higher in the outcome group with statistical significance (*p* = 0.002, *p* < 0.001, and *p* < 0.001, respectively). Among the mental disorders, anxiety disorder, neurosis, organic mental disorder, and adjustment disorder were more frequent in the outcome group with statistical significance (*p* < 0.001, *p* < 0.001, *p* < 0.001, and *p* = 0.012, respectively). In the case of nutritional disorder, undernutrition was more prevalent in the outcome group with statistical significance (*p* < 0.001). Among cardiovascular medication history, aspirin, antiplatelet agent, angiotensin-converting enzyme inhibitors, non-selective beta-blocker, and verapamil/diltiazem were more frequent in the outcome group with statistical significance (all *p* < 0.001). The selective beta-blocker was significantly lower in the outcome group (*p* < 0.001).

### 3.2. Variables

**Figure 1:**
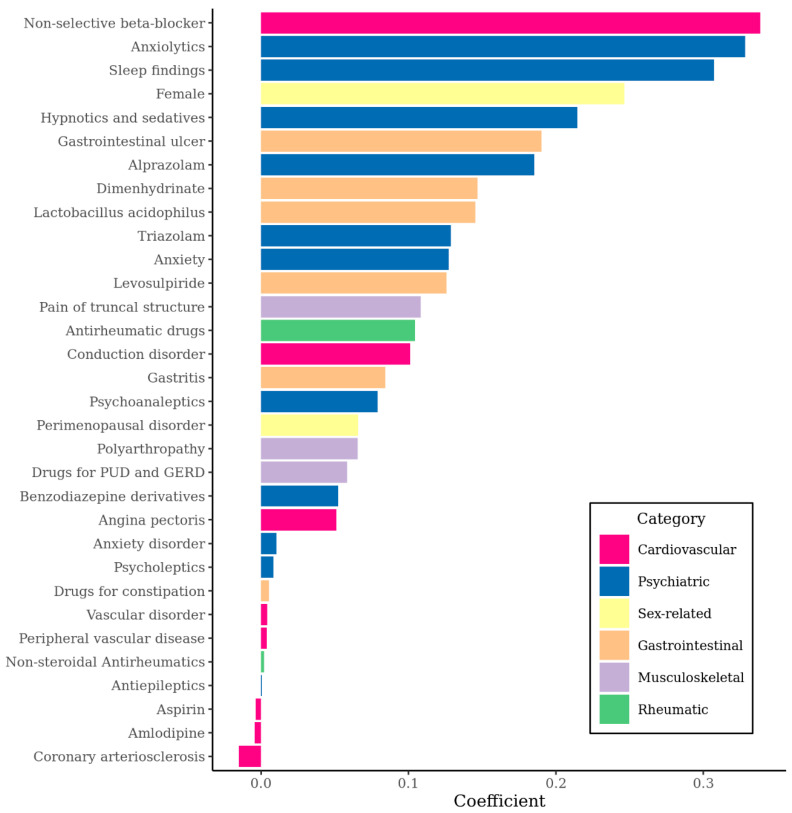
Coefficients of variables included in the developed model, as predictors for incident major depressive disorder. The higher coefficient means more predictive. PUD: peptic ulcer disease; GERD: gastro-esophageal reflux disease.

To train the *L*1 regularized logistic regression model, the NHIS-NSC database was converted to a total of 10,004 variables including clinical information about age, sex, diagnosis, drug, and procedure from about one million individuals. Among them, 74 variables were selected by *L*1 regularization and were included in the final model. Some of these variables are listed in Figure 1.

Among 74 variables finally selected in the model, the non-selective beta-blocker was the predictor with the highest coefficient (coefficient = 0.34). The following high coefficient predictors were anxiolytics, sleep findings, and female sex. Conditions and drugs associated with psychiatric disorders were included in the model such as sedatives, alprazolam, triazolam, anxiety, and benzodiazepines. Also, factors related to chronic disease such as gastrointestinal, musculoskeletal, and rheumatic chronic disorders were also predictors with high coefficients. Among CVDs used as the indication of a beta-blocker, angina pectoris was most predictive to MDD development and coronary arteriosclerosis was most predictive to not developing MDD. A full list of 74 variables selected as predictors of the model and their standardized mean difference is shown in Supplementary Table S5.

### 3.3. Model Performance

**Figure 2:**
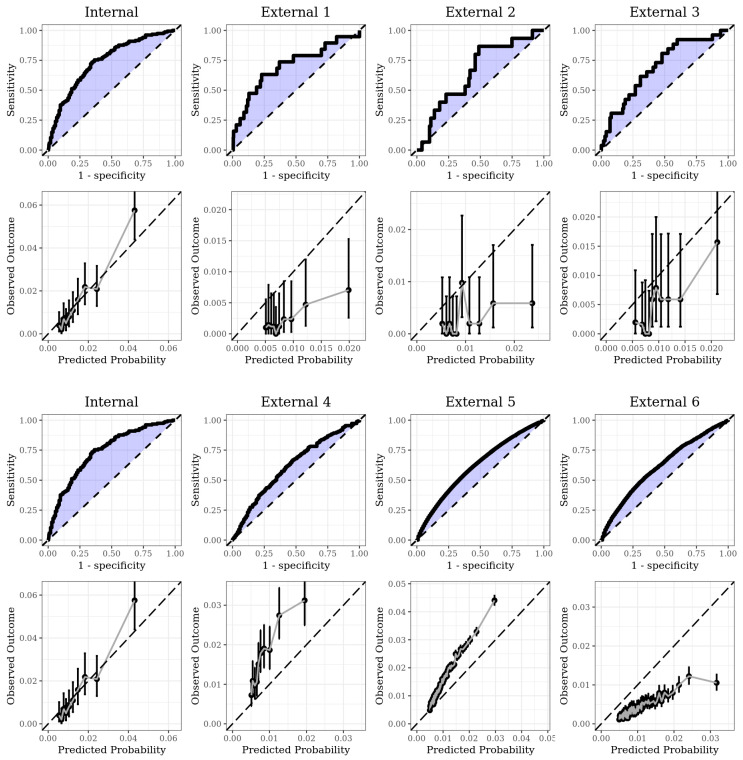
The area under receiver operating characteristic (AUROC) plot showing the performance of the *L*1 regularized logistic regression model and calibration plot comparing predicted probability calculated by the prediction model with the fraction of observed outcome.

A total of 154 (1.53%) MDD outcome events from 10,078 beta-blockers users in internal validation were obtained, with an AUC value of 0.74. Sensitivity and specificity were 83.1% and 49.5%, respectively. The performance of the model in internal and external validations is listed in Table 2. In six external validations, the number of outcomes was 19, 15, 26, 439, 59045, and 3342, respectively. AUCs were 0.71, 0.66, 0.70, 0.62, 0.62, and 0.62, each. Incidence ranged from 0.22% to 1.67%, some are much lower than internal validation data. Sensitivity and specificity were 78.9% and 49.0% in External 1, 86.7% and 49.4% in External 2, 80.8% and 49.9% in External 3, 77.2% and 40.4% in External 4, 75.1% and 40.2% in External 5, and 75.4% and 40.1% in External 6. The receiver operating characteristic (ROC) curve plot and calibration plot of each validation set are listed in Figure 2. Some of the ROC curves are rough due to the small number of outcomes causing a decrease of AUC. However, sensitivity and specificity were relatively retained comparing with internal validation.

Calibration plots depict the proportion between predicted probability calculated by the prediction model and the fraction of true observed outcome. In internal validation, we found that predicted risk and real observed outcome was proportional in a linear manner. In three external validations using the database from South Korea, the confidence interval was wide due to low frequency of outcome. However, a proportional tendency between predicted risk and fraction of outcome was still comparable. In three external validations using the databases from the USA, the proportion of observed outcome and predicted probability were proportional with a narrow confidence interval.

**Figure 3:**
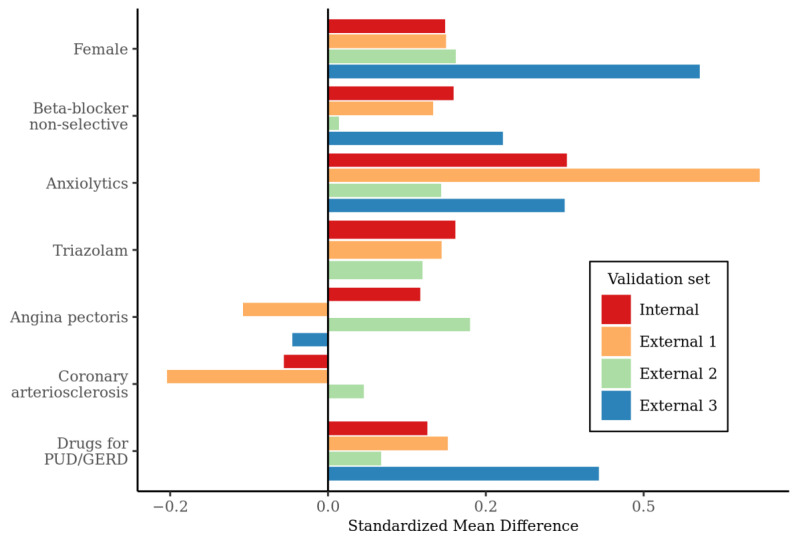
The comparison of the standardized mean differences of the predictors of major depressive disorder across databases in which validation is done. Positive number of standardized mean difference means that the variable is more frequent in outcome group than non-outcome group. PUD, peptic ulcer disease, GERD, gastro-esophageal reflux disease.

Furthermore, comparison of the standardized mean difference of the predictors, across internal and South Korean validation sets, was conducted and is shown in Figure 3. The following were consistently higher in the outcome group than in the non-outcome group across validation sets: female sex, non-selective beta-blocker, anxiolytics, triazolam, and drugs for peptic ulcer disease or gastro-esophageal reflux disease (GERD). Among variables showing a consistent difference, anxiolytics had the highest standardized mean difference in internal and external 1 validation sets (0.38, 0.69) and female sex had the highest standardized mean difference in external 2 and external 3 (0.20, 0.59). However, the incidence of angina pectoris and coronary arteriosclerosis were inconsistent across databases, although they were selected to be predictive in the model.

We observed that the general prescription of beta-blockers is decreasing in both selectivity and lipophilicity. The number of beta-blocker prescriptions by its selectivity and lipophilicity is listed in Supplementary Table S6. Furthermore, we calculated the incidence of MDD in patients with a history of anxiolytics and non-selective beta-blockers, which are the predictors with a high coefficient in the prediction model. While patients with no history of anxiolytics and no history of non-selective beta-blockers had only 0.8% of incident MDD, incidence increased to 2.5% and 3.4% in patients with a history of anxiolytics or non-selective beta-blockers, respectively. For patients with a history of both anxiolytics and non-selective beta-blockers, the incidence rate was 4.3%. The number of patients with a different history is listed in Supplementary Table S7.

## 4. Discussion

An ML model predicting incident MDD in patients initiating beta-blocker therapy for CVD was developed on the nationwide longitudinal claims database. The robustness of model performance was validated on multiple independent EHR and claims databases from South Korea and the USA. To the best of our knowledge, this is the first research utilizing extensive clinical data for discovering predictors of incident MDD in cardiovascular patients with beta-blockers. We identified various predictors including non-selective beta-blockers, anxiety disorder, sleep disorder, and chronic cardiovascular, musculoskeletal, and rheumatoid diseases.

Although we do not argue that there is independent causal relationship between use of non-selective beta-blockers and the risk of incident MDD in patients with CVD, the exposure of non-selective beta-blocker was the predictor with the highest coefficient value in our model. This aligns with a previous study that reported non-selective beta-blockers induce more depressive symptoms than other hypertensives [28]. There is still controversy as to whether beta-blocker induce depression in cardiovascular patients [29,30]. Mostly, old papers appear to be relevant, and relatively new ones are not [31]. This inconsistency might be due to the dilution of the effects of non-selective beta-blockers by various types of beta-blocker developed over time. Since the newer beta-blockers are mostly moderately lipophilic or hydrophilic, there is a possibility that overall association between beta-blockers and depression became weak.

Variables related to anxiety disorder were also strong predictors. Anxiety was often comorbid with depression [32], and there is a previous study that presented anxiety is a risk factor of depression [33]. Since anxiety is associated with poor cardiovascular outcome in patients with acute coronary syndrome, more vigilance is needed about comorbid depression and anxiety disorder [34]. We found that the incidence of MDD is numerically higher in patients with both a history of anxiolytics and the use of non-selective beta-blockers than patients with only one or none of these predictors.

Variables related to chronic cardiovascular, musculoskeletal, and rheumatoid diseases were selected as predictors. This is consistent with previous studies that showed a relationship between these diseases and depression [35,36]. As shown in the baseline characteristics of the study population, the prevalence of chronic diseases is not negligible so that the chance of a cardiovascular patient having other chronic diseases should be carefully examined. In addition, female sex was also a strong predictor of MDD. A previous study found that the lifetime prevalence of major depressive episodes was higher in females than in males and the onset was earlier in females [37]. This difference may arise from behavioral characteristics and fundamental genetic reasons [38,39]. In addition, perimenopausal disorder may contribute to the female sex being predictive of MDD, as shown in our model and a previous study [40].

This study has several limitations. First, the use of non-selective and lipophilic beta-blockers might decrease over time, although it was not evident in our development database. Second, outcomes reflecting depression severity were not included in the model due to limitation of the datasets. Not only onset, but also disease severity prediction is important regarding the proportional relationship between depression severity and poor prognosis [1]. However, this model predicted the onset of depression with only existing clinical data, unlike other studies that used variables that need to be newly measured or assessed [17,18]. Third, using only a diagnosis code for defining outcome event is another limitation. Nonetheless, this study used clearly specified standardized codes which is compatible with various code systems worldwide, and a previous study that showed the validity of defining depression using a code system in NHIS-NSC [41].

## 5. Conclusions

We developed a prediction model for incident MDD following long-term beta-blocker use in patients with CVDs. Variables including use of non-selective beta-blockers, female sex, history of anxiety, and common chronic diseases, such as gastrointestinal and musculoskeletal diseases, were predictive.

## Figures and Tables

**Table 1:** Basic characteristics of study population with outcome, and non-outcome.

Characteristics	Outcome (*n* = 774)	Non-Outcome (*n* = 49,623)	*p*-Value
Age (years, mean ± standard deviation (SD))	61.2 ± 12.9	58.7 ± 13.1	0.510
Male ^1^	298 (38.5)	25,527 (51.4)	<0.001
Beta-blocker indication ^2^			
Hypertensive disorder	726 (93.8)	47,321 (95.4)	0.050
Myocardial infarction	46 (5.9)	2456 (4.9)	0.238
Angina pectoris	205 (26.5)	8899 (17.9)	<0.001
Coronary arteriosclerosis	13 (1.7)	1592 (3.2)	0.021
Heart failure	98 (12.7)	4688 (9.4)	0.003
Chronic disease ^3^			
Cancer	24 (3.1)	1056 (2.1)	0.084
Chronic lung disease	98 (12.7)	4621 (9.3)	0.002
Stroke	45 (5.8)	1441 (2.9)	<0.001
Alzheimer’s disease	7 (0.9)	114 (0.2)	<0.001
Diabetes	135 (17.4)	9691 (19.5)	0.159
Chronic kidney disease	12 (1.6)	490 (1.0)	0.167
Mental disorder			
Anxiety disorder	150 (14.9)	3911 (7.9)	<0.001
Neurosis	62 (8.0)	1731 (3.5)	<0.001
Organic mental disorder	30 (3.9)	603 (1.2)	<0.001
Adjustment disorder	6 (0.8)	116 (0.2)	0.012
Personality disorder	0 (0.0)	60 (0.1)	1.000
Delusional disorder	1 (0.1)	14 (0.0)	0.207
Nutritional disorder			
Vitamin deficiency	9 (1.2)	301 (0.6)	0.08
Undernutrition	16 (2.1)	371 (0.7)	<0.001
Medication ^3^			
VKA	13 (1.7)	599 (1.2)	0.305
Aspirin	304 (39.3)	10,990 (22.1)	<0.001
Antiplatelet agents	66 (8.5)	2604 (5.2)	<0.001
ACEi	165 (21.5)	4795 (9.7)	<0.001
Angiotensin II receptor blocker	233 (30.1)	14,926 (30.1)	1.000
Selective beta-blocker	556 (71.8)	37,234 (75.0)	0.046
Non-selective beta-blocker	375 (48.4)	17,768 (35.8)	<0.001
Hydrophilic beta-blocker	543 (71.8)	36,302 (73.2)	0.068
Lipophilic beta-blocker	388 (50.1)	18,700 (37.7)	<0.001
Diuretic	400 (51.7)	25,985 (52.4)	0.732
Calcium channel antagonist	415 (53.6)	28,057 (56.5)	0.112
Cardiac glycoside	26 (3.4)	1575 (3.2)	0.851
Aldosterone antagonist	52 (6.7)	2846 (5.7)	0.277
Verapamil/diltiazem	80 (10.3)	3204 (6.5)	<0.001
Antiarrhythmics	14 (1.8)	517 (1.0)	0.058
Other immunosuppressants ^4^	1 (0.1)	153 (0.3)	0.735
Calcineurin inhibitors	4 (0.5)	132 (0.3)	0.157
Selective immunosuppressants	1 (0.1)	78 (0.2)	1.000
Tumor necrosis factor alpha -inhibitor	0 (0.0)	2 (0.0)	1.000

Number of persons were presented as number (percent in outcome or non-outcome group), except age. The chi-squared test was used for comparison between categorical variables, and the Fisher’s exact test was used for the categorical variables with frequency less than 5. The *t*-test was used for continuous variables. Statistical significance was defined as a *p*-value of <0.05 in a two-tailed test. ^1^ Mean of continuous variables were presented as mean (standard deviation). ^2^ Diagnosis record of cardiovascular disease at the time of beta-blocker prescription were counted and each diagnosis is not exclusive. ^3^ Diagnosis or drug exposure history in one year prior to index date were counted. VKA, vitamin K antagonist; ACEi, angiotensin converting enzyme inhibitors. ^4^ Other immunosuppressants include methotrexate, azathioprine, and thalidomide.

**Table 2:** Performance of the model in internal and external validations.

Validation Set	Name	*n*	Outcome	Incidence (%)	AUC	Sensitivity	Specificity
Internal	NHIS	10,078	154	1.53	0.74	83.1%	49.5%
External 1	Ajou	8511	19	0.22	0.71	78.9%	49.0%
External 2	Hanyang	5112	15	0.29	0.66	86.7%	49.4%
External 3	Kandong	5097	26	0.51	0.70	80.8%	49.9%
External 4	STARR	26,258	439	1.67	0.62	77.2%	40.4%
External 5	OpenClaims	4,295,013	59,045	1.38	0.62	75.1%	40.2%
External 6	AmbEMR	883,198	3342	0.38	0.62	75.4%	40.1%

AUC, area under the receiver operating characteristic curve.

## Supplementary Materials

The following are available online at https://www.mdpi.com/2075-4426/10/4/288/s1, Table S1: Description of the USA databases used in validation, Table S2: The concepts and logics used to define the target cohort and outcome event, Table S3: The settings and results of experimental models, Table S4: Number of patients and outcome incidence by age group, Table S5: Full list of variables selected in the final model, Table S6: The number of beta-blocker prescriptions by its selectivity and lipophilicity, Table S7: Number of patients and outcome incidence by history records.
