# Supplementary material for: Prediction of Major Depressive Disorder Following Beta-Blocker Therapy in Patients with Cardiovascular Diseases
Source: J Pers Med. 2020 Dec 18;10(4):288. doi: 10.3390/jpm10040288 (PMC7766565; doi:10.3390/jpm10040288)
Supplement: Supplementary file 1 [file jpm-10-00288-s001.zip › Supplementary Table S1.docx]

**Supplementary Table S1.** Description of the USA databases used in validation.

| **Database name** | **Description** |
| --- | --- |
| IQVIA Ambulatory EMR | The IQVIA US Ambulatory EMR database consists of longitudinal, de-identified electronic health records originating from ambulatory clients spanning from January 2006 to May 2020. The data contains detailed clinical information that captures important health outcomes such as lab test results and vital signs. It also covers administered drugs including prescription and over-the-counter medicines, vaccines, large-molecule biologic therapies, route of administration, days suppl and refill information |
| IQVIA Open Claims | IQVIA Open Claims database consists of open, pre-adjudicated medical (inpatient and outpatient) and pharmacy claims from January 2013 to May 2020. These data cover more than a 200 million unique patients with over 1 billion medical claims, 3 billion service claims and 4 billion prescription claims obtained annually from 800,000+ healthcare providers (~75% of physicians certified by the American Medical Association). |
| STARR-OMOP | STAnford medicine Research data Repository (STARR), a clinical data warehouse contains live Epic data from Stanford Health Care, the Stanford Children’s Hospital, the University Healthcare Alliance and Packard Children's Health Alliance clinics and other auxiliary data from Hospital applications such as radiology PACS. STARR-OMOP is our standardized database within STARR that has been converted into the OMOP-CDM version 5.3.1. STARR-OMOP contains EHR data for over 3 million patients from July 2008 to November 2020. STARR platform is developed and operated by Stanford Medicine Research IT team and is made possible by Stanford School of Medicine Research Office. |

EMR: electronic medical record, OMOP: Observational Medical Outcomes Partnership.
