# Supplementary material for: Prediction of Major Depressive Disorder Following Beta-Blocker Therapy in Patients with Cardiovascular Diseases
Source: J Pers Med. 2020 Dec 18;10(4):288. doi: 10.3390/jpm10040288 (PMC7766565; doi:10.3390/jpm10040288)
Supplement: Supplementary file 1 [file jpm-10-00288-s001.zip › Supplementary Table S2.docx]

**Supplementary Table S2.** The concepts and logics used to define the target cohort and outcome event.

**Logics to define the target cohort**

Initial Event Cohort

- People having any of the following:
  - a drug era of cardiovascular beta-blockers^1^
    - with era length >= 30
    - with age in years at era start >= 18
- with continuous observation of at least 365 days prior and 0 days after event index date, and limit initial events to: **earliest event per person.**

Inclusion Rules

Inclusion Criteria #1: cardiovascular indication

- Having all of the following criteria:
  - at least 1 occurrences of a condition occurrence of cardiovascular indication^2^ where events start between 3 days Before and 3 days After index start date

Inclusion Criteria #2: no PHx. of selected mental disorders

- Having all of the following criteria:
  - exactly 0 occurrences of a condition occurrence of depressive disorder^3^ where event starts between all days Before and 0 days After index start date
  - exactly 0 occurrences of a condition occurrence of schizophrenia^4^ where event starts between all days Before and 0 days After index start date

Inclusion Criteria #3: no PHx. of ANTIDEPRESSANTS

- Having all of the following criteria:
  - exactly 0 occurrences of a drug exposure of ANTIDEPRESSANTS^5^ where event starts between all days Before and 0 days After index start date

**Concepts to define the target cohort**

1. Cardiovascular beta-blocker

| INVALID_REASON_CAPTION | CONCEPT_ID | CONCEPT_CODE | CONCEPT_NAME | DOMAIN_ID | STANDARD_CONCEPT_CAPTION | CONCEPT_CLASS_ID | INVALID_REASON | isExcluded | includeDescendants |
| --- | --- | --- | --- | --- | --- | --- | --- | --- | --- |
| Valid | 1319998 | 149 | Acebutolol | Drug | Standard | Ingredient | V | FALSE | TRUE |
| Valid | 1314002 | 1202 | Atenolol | Drug | Standard | Ingredient | V | FALSE | TRUE |
| Valid | 40019430 | 371065 | Betaxolol Oral Tablet | Drug | Standard | Clinical Drug Form | V | FALSE | TRUE |
| Valid | 1338005 | 19484 | Bisoprolol | Drug | Standard | Ingredient | V | FALSE | TRUE |
| Valid | 1346823 | 20352 | carvedilol | Drug | Standard | Ingredient | V | FALSE | TRUE |
| Valid | 19063580 | 49737 | esmolol | Drug | Standard | Ingredient | V | FALSE | TRUE |
| Valid | 1386957 | 6185 | Labetalol | Drug | Standard | Ingredient | V | FALSE | TRUE |
| Valid | 1307046 | 6918 | Metoprolol | Drug | Standard | Ingredient | V | FALSE | TRUE |
| Valid | 1313200 | 7226 | Nadolol | Drug | Standard | Ingredient | V | FALSE | TRUE |
| Valid | 1314577 | 31555 | nebivolol | Drug | Standard | Ingredient | V | FALSE | TRUE |
| Valid | 1327978 | 7973 | Penbutolol | Drug | Standard | Ingredient | V | FALSE | TRUE |
| Valid | 1345858 | 8332 | Pindolol | Drug | Standard | Ingredient | V | FALSE | TRUE |
| Valid | 1353766 | 8787 | Propranolol | Drug | Standard | Ingredient | V | FALSE | TRUE |
| Valid | 1370109 | 9947 | Sotalol | Drug | Standard | Ingredient | V | FALSE | TRUE |
| Valid | 40087920 | 374135 | Timolol Oral Tablet | Drug | Standard | Clinical Drug Form | V | FALSE | TRUE |

2. Cardiovascular indication

| INVALID_REASON_CAPTION | CONCEPT_ID | CONCEPT_CODE | CONCEPT_NAME | DOMAIN_ID | STANDARD_CONCEPT_CAPTION | CONCEPT_CLASS_ID | INVALID_REASON | isExcluded | includeDescendants |
| --- | --- | --- | --- | --- | --- | --- | --- | --- | --- |
| Valid | 316866 | 38341003 | Hypertensive disorder | Condition | Standard | Clinical Finding | V | FALSE | TRUE |
| Valid | 321318 | 194828000 | Angina pectoris | Condition | Standard | Clinical Finding | V | FALSE | TRUE |
| Valid | 4329847 | 22298006 | Myocardial infarction | Condition | Standard | Clinical Finding | V | FALSE | TRUE |
| Valid | 316139 | 84114007 | Heart failure | Condition | Standard | Clinical Finding | V | FALSE | TRUE |
| Valid | 317576 | 53741008 | Coronary arteriosclerosis | Condition | Standard | Clinical Finding | V | FALSE | TRUE |

3. Depressive disorder

| INVALID_REASON_CAPTION | CONCEPT_ID | CONCEPT_CODE | CONCEPT_NAME | DOMAIN_ID | STANDARD_CONCEPT_CAPTION | CONCEPT_CLASS_ID | INVALID_REASON | isExcluded | includeDescendants |
| --- | --- | --- | --- | --- | --- | --- | --- | --- | --- |
| Valid | 440383 | 35489007 | Depressive disorder | Condition | Standard | Clinical Finding | V | FALSE | TRUE |

4. Schizophrenia

| INVALID_REASON_CAPTION | CONCEPT_ID | CONCEPT_CODE | CONCEPT_NAME | DOMAIN_ID | STANDARD_CONCEPT_CAPTION | CONCEPT_CLASS_ID | INVALID_REASON | isExcluded | includeDescendants |
| --- | --- | --- | --- | --- | --- | --- | --- | --- | --- |
| Valid | 435783 | 58214004 | Schizophrenia | Condition | Standard | Clinical Finding | V | FALSE | TRUE |

5. Antidepressants

| INVALID_REASON_CAPTION | CONCEPT_ID | CONCEPT_CODE | CONCEPT_NAME | DOMAIN_ID | STANDARD_CONCEPT_CAPTION | CONCEPT_CLASS_ID | INVALID_REASON | isExcluded | includeDescendants |
| --- | --- | --- | --- | --- | --- | --- | --- | --- | --- |
| Valid | 21604690 | N06A | ANTIDEPRESSANTS | Drug | Classification | ATC 3rd | V | FALSE | TRUE |

**Logics to define outcome event**

Initial Event Cohort

- People having any of the following:
  - A condition occurrence of Major Depressive Disorder^1^
    - For the first time in the person’s history
- with continuous observation of at least 0 days prior and 0 days after event index date, and limit initial events to: **all events per person.**

**Concepts to define outcome event**

1. Major Depressive Disorder

| INVALID_REASON_CAPTION | CONCEPT_ID | CONCEPT_CODE | CONCEPT_NAME | DOMAIN_ID | STANDARD_CONCEPT_CAPTION | CONCEPT_CLASS_ID | INVALID_REASON | isExcluded | includeDescendants |
| --- | --- | --- | --- | --- | --- | --- | --- | --- | --- |
| Valid | 4152280 | 370143000 | Major depressive disorder | Condition | Standard | Clinical Finding | V | FALSE | TRUE |
