# Supplementary material for: Prediction of Major Depressive Disorder Following Beta-Blocker Therapy in Patients with Cardiovascular Diseases
Source: J Pers Med. 2020 Dec 18;10(4):288. doi: 10.3390/jpm10040288 (PMC7766565; doi:10.3390/jpm10040288)
Supplement: Supplementary file 1 [file jpm-10-00288-s001.zip › Supplementary Table S3.docx]

**Supplementary Table S3.** The settings and results of experimental models.

**Lasso Logistic Regression Settings**

| Covariates | Settings |
| --- | --- |
| seed |  |
| variance | 0.04 |

**Random Forest Settings**

| Covariates | Settings |
| --- | --- |
| maxDepth | 4, 10, 17 |
| varImp | TRUE |

**Gradient Boosting Machine Settings**

| Covariates | Settings |
| --- | --- |
| maxDepth | 4, 6, 17 |
| minRows | 20 |
| seed |  |
| nthread | 20 |
| ntrees | 10, 100 |
| learnRate | 0.01, 0.1 |

**Performance**

| **Model** | **AUC** | **Sensitivity** | **Specificity** | **No. of Variables** |
| --- | --- | --- | --- | --- |
| Lasso logistic Regression | 0.67 | 74.7% | 49.4% | 58 |
| Random forest | 0.69 | 76.2% | 49.4% | 2597 |
| Gradient boosting machine | 0.69 | 75.7% | 49.4% | 121 |
