# Supplementary material for: Prediction of Major Depressive Disorder Following Beta-Blocker Therapy in Patients with Cardiovascular Diseases
Source: J Pers Med. 2020 Dec 18;10(4):288. doi: 10.3390/jpm10040288 (PMC7766565; doi:10.3390/jpm10040288)
Supplement: Supplementary file 1 [file jpm-10-00288-s001.zip › Supplementary Table S4.docx]

**Supplementary Table S4.** Number of patients and outcome incidence by age group.

| **Age group** | **Number** | **Incidence (%)** | **Male (%)** | **Female (%)** |
| --- | --- | --- | --- | --- |
| Age < 30 | 567 | 2.12 | 1.41 | 0.71 |
| 30 <= Age < 40 | 2,760 | 0.94 | 0.51 | 0.43 |
| 40 <= Age < 50 | 9,304 | 1.31 | 0.60 | 0.71 |
| 50 <= Age < 60 | 13,163 | 1.30 | 0.51 | 0.79 |
| 60 <= Age < 70 | 13,690 | 1.59 | 0.59 | 1.00 |
| 70 <= Age < 80 | 8,359 | 2.05 | 0.65 | 1.40 |
| 80 <= Age | 2,554 | 2.11 | 0.70 | 1.41 |
