# Supplementary material for: Prediction of Major Depressive Disorder Following Beta-Blocker Therapy in Patients with Cardiovascular Diseases
Source: J Pers Med. 2020 Dec 18;10(4):288. doi: 10.3390/jpm10040288 (PMC7766565; doi:10.3390/jpm10040288)
Supplement: Supplementary file 1 [file jpm-10-00288-s001.zip › Supplementary Table S5.docx]

**Supplementary Table S5.** Full list of variables selected in the final model.

| **Variable name** | **Concept id** | **Coefficient** | **Standardized mean diff** |
| --- | --- | --- | --- |
| ALIBENDOL | 19055880 | -0.02846279 | 0.078518544 |
| Alprazolam | 781039 | 0.185331854 | 0.250531567 |
| Amlodipine | 1332418 | -0.00440318 | -0.04710383 |
| Angina pectoris | 321318 | 0.051174449 | 0.146216996 |
| ANTI-DEMENTIA DRUGS | 21604792 | 0.067211426 | 0.160905761 |
| ANTIBIOTICS AND CHEMOTHERAPEUTICS FOR DERMATOLOGICAL USE | 21602054 | 0.054321018 | 0.174276915 |
| ANTIEPILEPTICS | 21604390 | 0.000433989 | 0.143680316 |
| ANTIEPILEPTICS | 21604389 | 0.025617253 | 0.143680316 |
| ANTIINFLAMMATORY AND ANTIRHEUMATIC PRODUCTS | 21603932 | 0.104496258 | 0.209916844 |
| ANTIINFLAMMATORY AND ANTIRHEUMATIC PRODUCTS, NON-STEROIDS | 21603933 | 0.001892717 | 0.209916844 |
| Anxiety | 441542 | 0.127185258 | 0.240256998 |
| Anxiety disorder | 442077 | 0.010400742 | 0.240256998 |
| ANXIOLYTICS | 21604564 | 0.328450435 | 0.378584234 |
| Aspirin | 1112807 | -0.0036137 | 0.024124524 |
| Backache | 134736 | 0.000678857 | 0.162629342 |
| Benzodiazepine derivatives | 21604565 | 0.052407679 | 0.370977811 |
| Beta blocking agents, non-selective | 21601666 | 0.338506105 | 0.198656576 |
| Cataract | 375545 | 0.090581224 | 0.129102097 |
| Cetirizine | 1149196 | 0.031835233 | 0.09264953 |
| Chlorpheniramine | 1192710 | -0.02339909 | 0.102877534 |
| Chronic care management services, at least 20 minutes of clinical staff time directed by a physician or other qualified health care professional, per calendar month, with the following required elements: multiple (two or more) chronic conditions expected | 46257536 | -0.00322729 | -0.00635205 |
| Chronic inflammatory disorder | 444208 | 0.098674705 | 0.218235945 |
| Conduction disorder of the heart | 316999 | 0.101063015 | 0.132591907 |
| Coronary arteriosclerosis | 317576 | -0.0152506 | -0.07007195 |
| Diabetes mellitus | 201820 | 0.053673963 | 0.108764082 |
| Difficulty breathing | 4041664 | 0.014110895 | 0.149564697 |
| Dimenhydrinate | 928744 | 0.146941746 | 0.187669295 |
| Dizziness | 4223938 | 0.014850367 | 0.193782061 |
| DRUGS FOR CONSTIPATION | 21600531 | 0.116288708 | 0.201899191 |
| DRUGS FOR CONSTIPATION | 21600532 | 0.005466935 | 0.201899191 |
| Dyspnea | 312437 | 0.032867739 | 0.149564697 |
| Emergency treatment management | 4295073 | 0.115355271 | 0.194346848 |
| Famotidine | 953076 | -0.00211312 | 0.10177324 |
| FEMALE | 8532 | 0.246649919 | 0.185456532 |
| Finding related to sleep | 444108 | 0.30729955 | 0.245478382 |
| Functional finding of respiratory tract | 4103331 | 0.08536514 | 0.13537862 |
| Gastritis | 201340 | 0.084153275 | 0.257850337 |
| Gastrointestinal ulcer | 4247120 | 0.190204875 | 0.248335423 |
| Giddiness | 4229392 | 0.070930802 | 0.192836445 |
| Hydrochlorothiazide | 974166 | -0.03923542 | -0.01793721 |
| HYPNOTICS AND SEDATIVES | 21604606 | 0.214474386 | 0.265782592 |
| Ibuprofen | 1177480 | -0.06205642 | 0.046568639 |
| Inflammation of specific body organs | 4181063 | 0.031973538 | 0.227694351 |
| Inflammatory disorder of genitourinary system | 4159963 | 0.050026972 | 0.166686128 |
| Insulins and analogues for injection, intermediate- or long-acting combined with fast-acting | 21600728 | 0.065427786 | 0.136701473 |
| Intravenous injection | 4181778 | 0.093605809 | 0.272414988 |
| Labyrinthine disorder | 81303 | 0.024058999 | 0.166774688 |
| Lactobacillus acidophilus | 987153 | 0.145449911 | 0.148568469 |
| levosulpiride | 43009023 | 0.125863315 | 0.224077653 |
| Magnesium compounds | 21600048 | 0.010029776 | 0.171760653 |
| Magnesium Hydroxide | 992956 | 0.016137708 | 0.166390176 |
| Mannitol | 994058 | 0.105092112 | 0.167663456 |
| Other antibiotics for topical use | 21602061 | 0.14191023 | 0.148559291 |
| OTHER BETA-LACTAM ANTIBACTERIALS | 21602868 | 0.044359828 | 0.169390124 |
| Other drugs for peptic ulcer and gastro-oesophageal reflux disease (GORD) | 21600109 | 0.058466527 | 0.15725766 |
| Pain of truncal structure | 4116811 | 0.108463811 | 0.231259285 |
| Perimenopausal disorder | 4141640 | 0.06590645 | 0.133298787 |
| Peripheral vascular disease | 321052 | 0.003974783 | 0.148236218 |
| Polyarthropathy | 75897 | 0.06559876 | 0.133172981 |
| Propionic acid derivatives | 21603966 | -0.02016103 | 0.082878585 |
| PSYCHOANALEPTICS | 21604685 | 0.079061522 | 0.173396938 |
| PSYCHOLEPTICS | 21604489 | 0.008245532 | 0.391482436 |
| Salicylic acid and derivatives | 21604304 | -0.00827061 | 0.025164949 |
| SENSORY ORGANS | 21603550 | 0.042175679 | 0.236589036 |
| Soft tissue lesion | 4344497 | 0.059812601 | 0.185195389 |
| Superficial mycosis | 4077081 | -0.08221585 | -0.03200639 |
| Synthetic anticholinergics, esters with tertiary amino group | 21600129 | 0.008359482 | 0.176385084 |
| Therapeutic radiology port film interpretation and verification | 4264890 | 0.30473612 | 0.185100374 |
| Thyrotoxicosis | 138387 | 0.012461401 | 0.117989276 |
| Traumatic injury | 440921 | 0.052302386 | 0.153567734 |
| Triazolam | 704599 | 0.128892527 | 0.202079245 |
| Urine examination | 4055811 | 0.017347766 | 0.209181994 |
| Vascular disorder | 443784 | 0.004051569 | 0.159029333 |
| VITAMIN B12 AND FOLIC ACID | 21601119 | 0.001775347 | 0.111549979 |
