# Supplementary material for: Prediction of Major Depressive Disorder Following Beta-Blocker Therapy in Patients with Cardiovascular Diseases
Source: J Pers Med. 2020 Dec 18;10(4):288. doi: 10.3390/jpm10040288 (PMC7766565; doi:10.3390/jpm10040288)
Supplement: Supplementary file 1 [file jpm-10-00288-s001.zip › Supplementary Table S6.docx]

**Supplementary Table S6.** The number of beta-blocker prescriptions by its selectivity and lipophilicity.

- Number of beta-blocker prescription by selectivity.

| **Selectivity** | **2003** | **2004** | **2005** | **2006** | **2007** | **2008** | **2009** | **2010** | **2011** | **2012** |
| --- | --- | --- | --- | --- | --- | --- | --- | --- | --- | --- |
| Non-selective | 2,537 | 2,140 | 1,983 | 1,923 | 1,656 | 1,972 | 1,620 | 1,506 | 1,661 | 1,136 |
| Selective | 11,042 | 6,206 | 5,216 | 3,376 | 2,566 | 2,351 | 1,877 | 1,668 | 1,991 | 1,489 |
| Total | 13,579 | 8,346 | 7,199 | 5,299 | 4,222 | 4,323 | 3,497 | 3,174 | 3,652 | 2,625 |
| Non-selective to selective ratio | 0.23 | 0.34 | 0.38 | 0.57 | 0.65 | 0.84 | 0.86 | 0.90 | 0.83 | 0.76 |

- Number of beta-blocker prescription by lipophilicity.

| **Lipophilicity** | **2003** | **2004** | **2005** | **2006** | **2007** | **2008** | **2009** | **2010** | **2011** | **2012** |
| --- | --- | --- | --- | --- | --- | --- | --- | --- | --- | --- |
| Lipophilic | 2,911 | 2,307 | 2,085 | 1,992 | 1,704 | 2,025 | 1,651 | 1,539 | 1,705 | 1,160 |
| Hydrophilic | 10,668 | 6,039 | 5,114 | 3,307 | 2,518 | 2,298 | 1,846 | 1,635 | 1,947 | 1,465 |
| Total | 13,579 | 8,346 | 7,199 | 5,299 | 4,222 | 4,323 | 3,497 | 3,174 | 3,652 | 2,625 |
| Lipophilic to hydrophilic ratio | 0.28 | 0.38 | 0.41 | 0.360 | 0.68 | 0.88 | 0.89 | 0.94 | 0.88 | 0.79 |
