# Supplementary material for: Prediction of Major Depressive Disorder Following Beta-Blocker Therapy in Patients with Cardiovascular Diseases
Source: J Pers Med. 2020 Dec 18;10(4):288. doi: 10.3390/jpm10040288 (PMC7766565; doi:10.3390/jpm10040288)
Supplement: Supplementary file 1 [file jpm-10-00288-s001.zip › Supplementary Table S7.docx]

**Supplementary Table S7.** Number of patients and outcome incidence by history records.

| History | Outcome (n) | Non-outcome (n) | Incidence (%) |
| --- | --- | --- | --- |
| None | 228 | 27,765 | 0.8 |
| Anxiolytics | 519 | 20,483 | 2.5 |
| Non-selective beta-blockers | 125 | 3,578 | 3.4 |
| Both | 98 | 2,203 | 4.3 |

n: the number of patients.
